# Precision Microbiome: A New Era of Targeted Therapy with Core Probiotics

**DOI:** 10.34133/research.0658

**Published:** 2025-03-26

**Authors:** Xiuyu Fang, Yuhao Wang, Hong Wei, Yuan Huang

**Affiliations:** ^1^ College of Animal Science and Technology, Northeast Agricultural University, Harbin 150030, China.; ^2^ Zhejiang Provincial Key Laboratory of Pancreatic Disease of The First Affiliated Hospital, Institute of Translational Medicine, Zhejiang University School of Medicine, Hangzhou 310029, Zhejiang, China.; ^3^ Yu-Yue Pathology Scientific Research Center, Jinfeng Laboratory, Chongqing 401329, China.; ^4^ State Key Laboratory of Cardiovascular Disease, Fuwai Hospital, National Center for Cardiovascular Diseases, Chinese Academy of Medical Sciences and Peking Union Medical College, Beijing 100037, China.

In recent years, the concept of “precision microbiome” has received a lot of attention from researchers. It involves the precise analysis and typing of microbiota in specific hosts (e.g., humans or animals) using advanced tools like high-throughput sequencing, genomics, and artificial intelligence (AI). These tools help explore the complex interactions between microbiota and hosts to provide more precise and personalized treatment strategies [1,2]. With the maturation of omics technologies and data analysis techniques, the essential role of gut microbiota in immune system maturation, barrier function maintenance, and metabolic regulation has become increasingly recognized [3]. However, the “hodgepodge” approach of traditional fecal microbiota transplantation (FMT) cannot be appropriately controlled in clinical applications. This challenge has spurred the emergence of targeted interventions focused on specific functional core probiotics. Unlike traditional FMT, this approach aims to precisely identify and target microbial functional genes and metabolic pathways, selecting core probiotics for application, to improve host health and prevent or manage diseases [4]. This article reviews the advantages and limitations of traditional FMT and core probiotic targeted therapy while exploring the future directions of precision microbiome research.

Microbes colonize the digestive tract shortly after birth and establish a symbiotic relationship with the host. A stable and balanced gut microbiota is essential for maintaining host health and controlling diseases [5]. Dysbiosis has been linked to various conditions, such as inflammatory bowel disease (IBD), metabolic disorders, and cancer. Advances in omics technologies have enabled a deeper understanding of the crucial relationship between the gut microbiota and host health [6]. Chinese documents in the 4th century recorded the use of FMT to treat food poisoning and diarrhea [7]. Since the early 20th century, microbiome-based interventions have been employed in clinical practice. Until 2013, with the publication of the first randomized controlled trial of FMT for recurrent *Clostridium difficile* infection (rCDI), the fecal microbiota products (FMPs) began to be systematically and commercially studied [8]. Research has led companies to expand FMP use to treat conditions such as IBD, graft-versus-host disease (GVHD), and cancer, broadening its clinical applications [9].

However, although FMT has shown initial effectiveness in treating various diseases, many factors can impact FMT. Currently, there remains significant disparity in treatment outcomes for conditions such as irritable bowel syndrome (IBS) and metabolic syndrome [10]. Different preparation methods, administration protocols, and delivery routes can also lead to large differences in experimental results. Some studies suggest that the effectiveness of FMT may depend on the successful cultivation of core probiotics in the microbiota [11]. Importantly, the safety of FMT cannot be ignored, as the standardization of healthy donors is difficult. Even after rigorous screening, underdetected pathogens may remain, such as antibiotic-resistant microbes, harmful viruses, proinflammatory metabolite-producing strains, and gastrointestinal fungi and viruses, which can have serious consequences, as demonstrated by the deaths of patients infected with drug-resistant bacteria [12]. Due to the uncertainty of the efficacy of FMT, it is important to validate the application of FMT against potential microbial infections when administered to immunocompromised patients.

Given the inherent limitations of traditional FMT, researchers have increasingly turned toward more controlled alternatives. Recent advancements in multi-omics technologies, particularly genomics and metabolomics, combined with AI-driven data analysis, have enabled precise identification and selection of core probiotics [13]. For example, the machine learning platform iProbiotics can facilitate rapid probiotic screening [14], and AI models with novel analytical technologies can further aid in identifying core members of the gut microbiota [15]. This targeted approach facilitates more precise, individualized treatments compared to traditional FMT, thereby bridging the narrative between limitations of conventional microbiota transplantation and next-generation precision microbiome therapeutics. In recent years, researchers have used known probiotics for clinical disease treatments, such as chronic constipation, Crohn’s disease, ulcerative colitis, depression, type 2 diabetes, rCDI, among others. Core probiotics are strains or groups of bacteria that play a key role in maintaining intestinal homeostasis and regulating specific diseases [16,17]. For instance, *Faecalibacterium prausnitzii* has been associated with IBD and its impact on gut mucosal immunity [18] and *Akkermansia muciniphila* has been related to obesity and metabolic syndrome [19], while *Lactobacillus murinus* has been linked to colitis and colorectal cancer [20]. The functions of these strains include not only short-chain fatty acid (SCFA) metabolism and regulation of immune cell subpopulations but also possible interaction with the host genome [21]. Additionally, this shift has led to the development of live biological products (LBPs), which consist of precisely defined combinations of single or multi-strain probiotics designed to reduce the risks associated with transplanting unknown pathogens or chronic diseases [22]. For example, studies have shown that multistrain probiotics applied for rCDI have the same effect as FMPs do, and a specific strain applied to IBS patients can significantly improve patients’ bowel habits. *Lactobacillus casei* isolated from Chinese fermented yoghurt samples can effectively slow down the decline of kidney function in patients with stage 3 to 5 chronic kidney disease [23]. The World Gastroenterology Organisation guidelines recommend *Lactobacillus reuteri* DSM17938 for the treatment of pediatric acute gastroenteritis, infant colic, functional abdominal pain in children, and prevention of necrotizing enterocolitis [24]. These studies suggest that LBPs can achieve similar or even better therapeutic effects compared to complex and potentially risky donor feces. Using known specific strains is a more refined approach and is more easily accepted by patients. Compared with FMT, LBPs are easier to standardize and have less batch-to-batch variation. Moreover, they can generally pass safety assessments with better results, and when applied clinically, they can provide at least one clear mechanism of action for different diseases or responses.

However, there are also several difficulties in expanding the application of LBPs. For example, before clinical application, the effects of strains are often validated in sterile rodent models of the target diseases. The microbiota commonly found in the human body may not be able to easily colonize mice, making it challenging to conduct preclinical validation trials. In addition, whether to use a single strain or a combination of multiple strains and what dosages to use need to be determined for different patients or conditions. The configuration and cultivation of multi-strain combination probiotics raise questions about how to ensure the viability of each strain and whether to culture them separately or together [25]. Furthermore, there is significant heterogeneity in lifestyles, dietary structures, and disease phenotypes among individuals. Determining how to evaluate the intervention effects of the same strain in a large population and how to dynamically adjust strain combinations requires more detailed prospective cohort studies. Owing to the limitations of existing culture conditions and technological methods, the application of known microbiota restricts the sources of potential probiotics, and strains that have not yet been isolated and cultivated are difficult to study and apply.

In the future, to better utilize precision microbiomes for targeted disease therapy, researchers can work to address the following challenges. First, the greatest challenge is to translate known preclinical results of probiotics into reliable clinical trials. Given the difficulty certain strains have in colonizing mice, we can attempt to use tools such as organ-on-a-chip, organoid, 3-dimensional (3D) cell culture, and AI models for toxicity prediction to further confirm the efficacy of the strains [26]. Second, safer and more effective LBP products should be designed. After understanding the effects and dosage of each strain, we can use metabolic models and AI to predict and design combinations of 2 or more strains, which may be more beneficial for strain colonization and restoring a healthy gut microecosystem [27]. When producing LBP products, efforts should also be made to minimize the oxygen exposure of strictly anaerobic bacteria, determine the optimal growth medium and physiological parameters, and ensure the viability and long-term stability of the strains. When applying core probiotics for targeted therapy, stratification of patients should be utilized to improve clinical design, as not all patients respond effectively to a given LBP. Biomarkers related to the microbiome should be established for precision therapy [28]. Moreover, efforts should be made to expand the selection of strains for LBP products to include strains that have not yet been cultured. Currently known strains represent only a small fraction of the entire gut microbiota. In recent years, advanced technologies such as machine learning and AI have greatly accelerated the identification and functional characterization of microbiota [29]. These computational tools can be used to predict microbial interactions, identify key microbial biomarkers, and optimize probiotic strain combinations. In addition, the use of innovative probiotic delivery systems, including microcapsules, targeted nanoparticles, and biocontrol agents, has shown great potential to improve the viability, stability, and targeted colonization of probiotics, resulting in greatly improved clinical efficacy [30,31]. Additionally, precision microbiome therapies may be greatly enhanced through integration with other cutting-edge technologies such as CRISPR-Cas9 gene editing and personalized nutrition. CRISPR-Cas9 can precisely modify probiotic genomes to enhance beneficial properties or eliminate harmful ones [32,33]. Personalized nutrition could further support microbiome interventions by tailoring dietary regimens to an individual’s microbiome profile, maximizing therapeutic efficacy and maintaining long-term microbial balance [34]. Finally, clinical research should be accompanied by proactive engagement with drug regulatory authorities to produce LBP products that target disease under the premise of meeting clinical application standards [35].

In addition, on the basis of research related to precision microbiomes, the use of postbiotics (the metabolic products of probiotics) may also be an effective method for achieving precision medicine goals [36]. Compared with the complex and difficult-to-control microbiota from FMT, the metabolites produced by strains allow better control of dosage and administration methods, making them potentially more effective and easier to use for clinical testing. Postbiotics contain various beneficial active components for the body, such as exopolysaccharides, SCFAs, lipoteichoic acid, vitamins, and antimicrobial peptides. For example, butyrate adjunct therapy may improve gut dysbiosis and enhance the quality of life of patients with ulcerative colitis [37]. Heat-inactivated *Lactobacillus plantarum* L-137 can clinically enhance immunity and ameliorate metabolic inflammation [38]. Therefore, in future precision microbiome treatment systems, the combined use of probiotics and postbiotics may represent a “two wings of a bird” model, better meeting the needs during different disease stages and progression [39].

In summary, the “precision microbiome” and “core probiotic targeted therapy” are not just extensions or improvements of FMT but rather new therapeutic approaches based on contemporary biotechnology and omics analysis. With this approach, researchers can use multiomics and big data mining to rapidly identify and cultivate microbiota or metabolites that have a decisive impact on certain diseases, making clinical interventions more targeted, safe, and replicable [40]. In the future, with the optimization of strain selection technology, improvements in fermentation cultivation processes, and the enrichment and improvement of postbiotics, this technology may bring about disruptive innovations in major disease areas such as chronic inflammation, tumors, and immune imbalance, providing a new foundation for precision medicine.
